# Aortopulmonary Fistulous Communication Between Stentless Root Xenograft and Homograft

**DOI:** 10.1016/j.atssr.2023.07.018

**Published:** 2023-08-19

**Authors:** Gabriel E. Cambronero, Elizabeth C. Wood, Kim M. Linden, Zohaib R. Khawaja, Yusuf M. Aboutabl, James E. Jordan, Neal D. Kon, Bartlomiej R. Imielski

**Affiliations:** 1 Department of Cardiothoracic Surgery, Wake Forest School of Medicine, Winston-Salem, North Carolina

## Abstract

Aortopulmonary fistulas are a rare complication of aortic root replacements associated with aortic aneurysms. We describe a patient with a sinus of Valsalva pseudoaneurysm associated with a Freestyle root replacement that fistulized to a distal pulmonary valve homograft after having 2 prior aortic and pulmonary valve surgeries. After a failed endovascular closure attempt, he underwent a third operation for repair.

Aortopulmonary fistulas (APFs) are a rare complication of aortic root replacement (ARR) and are often associated with aortic aneurysms. APFs are often quickly fatal, being discovered post mortem or during routine screening.^1^ Their cause is frequently attributed to pseudoaneurysmal erosion of the aorta into the pulmonary artery (PA).^2^ We describe a patient undergoing a third sternotomy to repair a sinus of Valsalva (SOV) pseudoaneurysm associated with a Freestyle (Medtronic) ARR that fistulized to a distal pulmonary valve homograft. Although SOV pseudoaneurysms and fistulas after Freestyle implantation have been described, this APF has formed between an aortic valve xenograft and a pulmonary valve homograft.

A 52-year-old man with a history of congenital aortic stenosis underwent a Ross procedure at the age of 25 years. By 49 years of age, severe aortic regurgitation developed with an aortic root aneurysm measuring 4.6 cm with a dilated ascending aorta, pulmonary valve regurgitation, and coronary artery disease for which a redo ARR with a 25-mm Freestyle using a Cabrol technique and hemiarch replacement, pulmonary valve replacement with a pulmonary homograft, and coronary artery bypass using a great saphenous vein graft to right coronary artery were performed. Three years later, he presented with dyspnea and “pounding” in his chest. His workup included transthoracic echocardiography, which identified an APF. Transesophageal echocardiography and computed tomography coronary angiography confirmed the findings of a left coronary sinus pseudoaneurysm measuring 16 by 12 mm with an APF communicating with the pulmonary homograft (Figure 1, Figure 2). A left-sided heart catheterization demonstrated mild coronary artery disease, a left coronary ostium that would remain unobstructed if endovascular closure were to be attempted, and elevated right-sided heart pressures consistent with mild pulmonary hypertension when the catheter crossed the APF into the PA.

**Figure 1:**
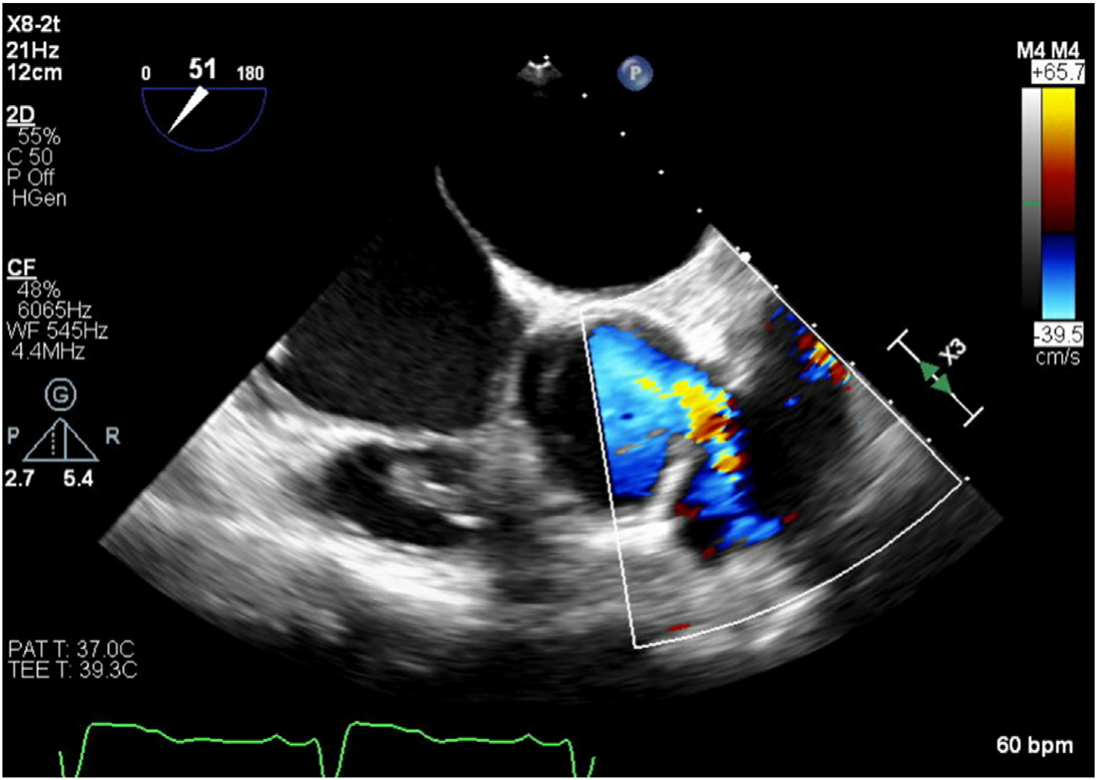
Transesophageal echocardiogram of the midesophageal right ventricle inflow-outflow window with color Doppler. Demonstrated is an aortopulmonary fistula near the left coronary cusp to the pulmonary artery with a left-to-right shunt.

**Figure 2:**
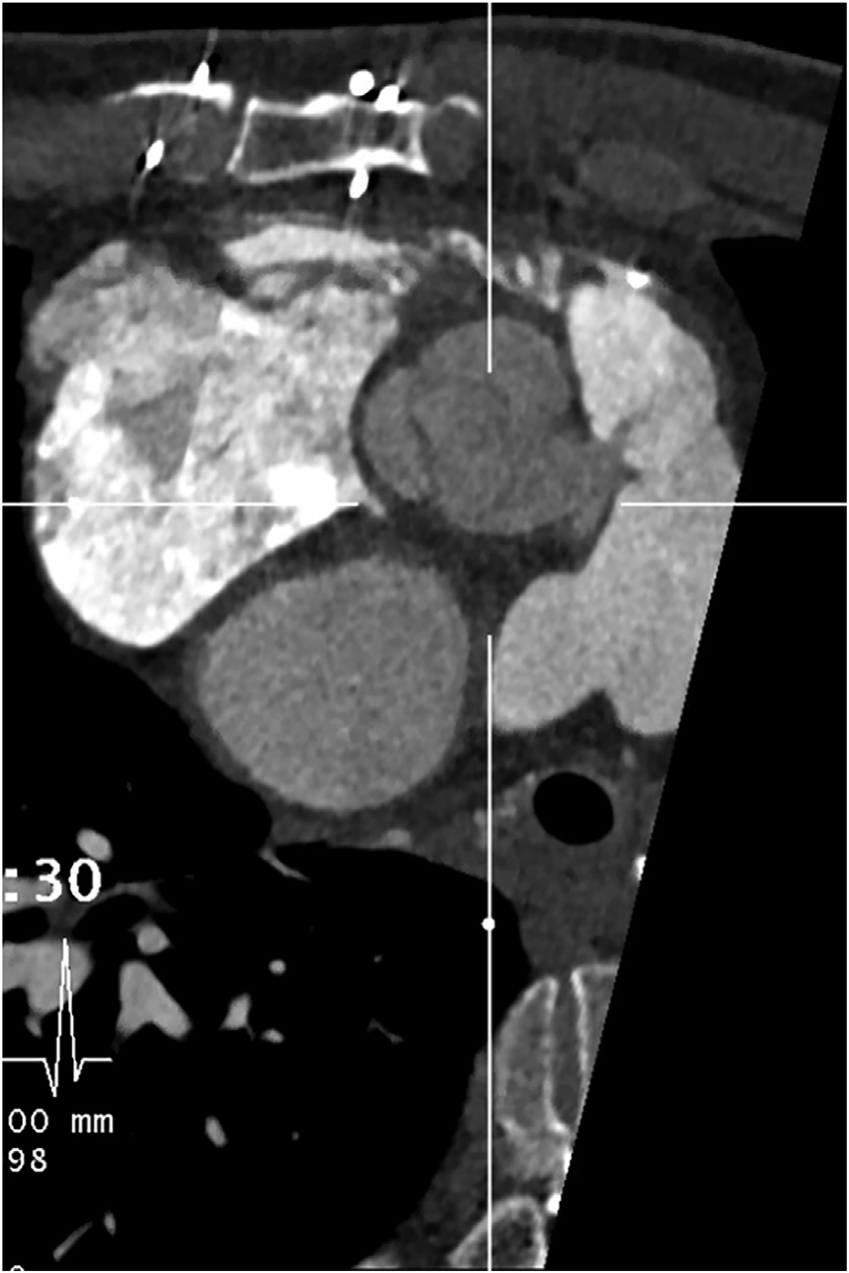
A pseudoaneurysm with a fistulous connection to the distal pulmonic valve homograft. Two distinct streams of contrast material are noted, with the fistula being just superior/anterior to the origin of the left main coronary artery.

Given negative blood cultures and no evidence of endocarditis, endovascular fistula closure was attempted using an 18-mm ventricular septal defect occlusion device. However, aortopulmonary flow could not be completely sealed. Therefore, the patient was scheduled for a third sternotomy. Repair of the APF with a bovine pericardial patch, redo ARR with a Freestyle graft with a Cabrol extension of the left main coronary artery (LMCA), and saphenous vein graft to the left anterior descending bypass were performed. The patient was cannulated peripherally for reoperative sternotomy. After safe entry, cardiopulmonary bypass was initiated, and the heart was arrested. The aortic graft was transected and the root inspected. Two perforations in the Freestyle bioprosthesis were found connecting to 1 larger hole in the PA. The first was above and next to the ostium of the LMCA; the second was in the tubular aorta above the sinotubular junction. There were no findings indicating infection. The decision was made to replace the entire aortic root and patch the pulmonary homograft. The Freestyle was excised sharply. Given reoperative scarring in the root, the LMCA had insufficient length after mobilization, and thus the previous short Cabrol graft was extended with a 7-mm vascular graft. The defect in the PA (Figure 3) was repaired using bovine pericardium and interrupted Prolene sutures. The new Freestyle was sized, implanted, and anastomosed to the distal ascending aorta. Weaning from cardiopulmonary bypass was complicated by postcardiotomy shock. Multiple interventions were performed to stabilize the patient, including a saphenous vein graft bypass to the distal left anterior descending artery should there have been any kinking of the Cabrol extension to the LMCA. It was thought that venous graft was safer than internal mammary graft in an already heparinized, unstable patient on bypass. An intra-aortic balloon pump was placed for hemodynamic support, and because of the right ventricular dysfunction, the chest was left open.

**Figure 3:**
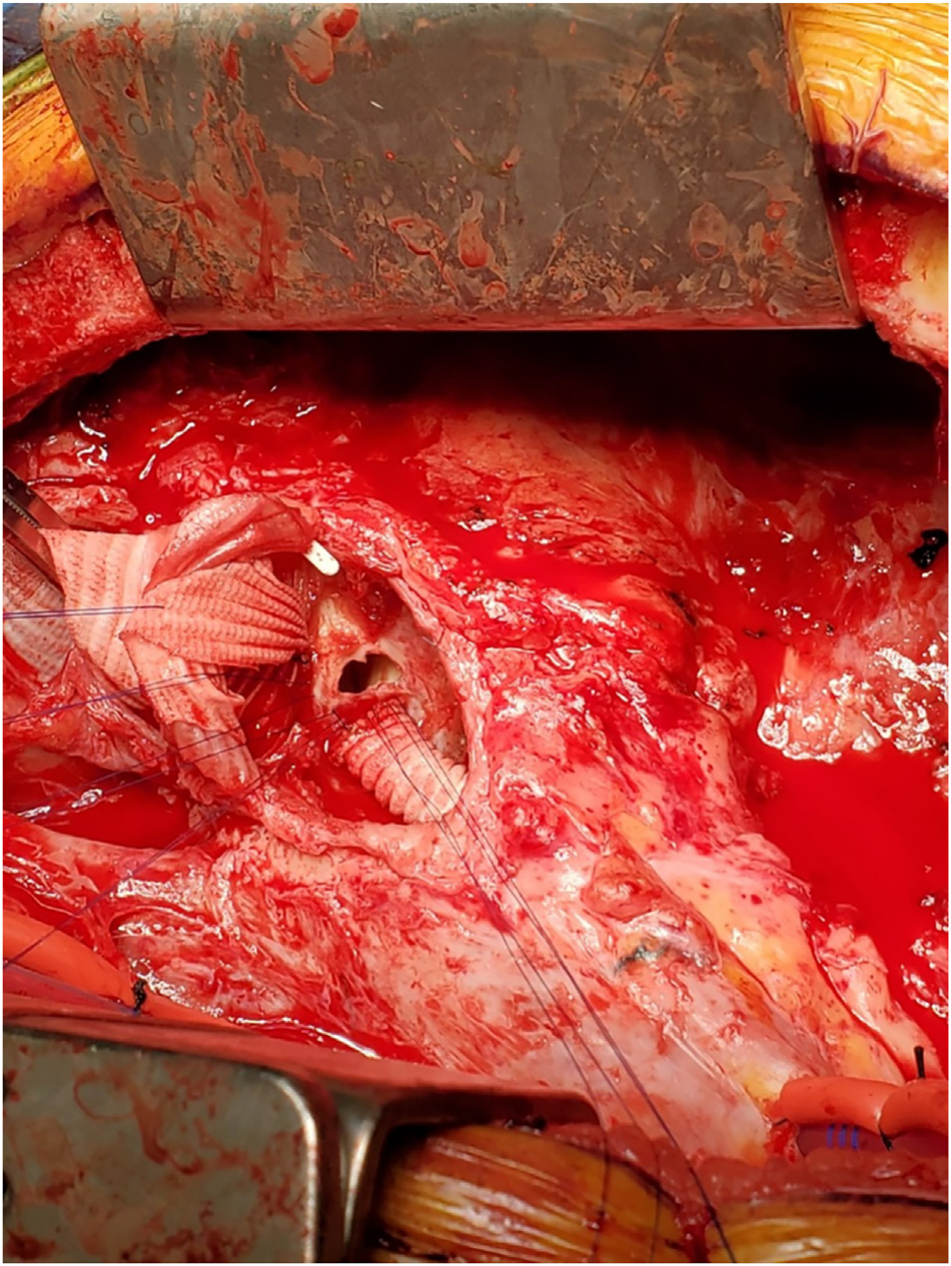
The aortopulmonary fistula as seen intraoperatively. Stay sutures retract the ascending aortic graft cephalad, revealing the fistula into the pulmonary artery. The Cabrol graft off the left coronary button is inferior to the fistula.

After resuscitation, his cardiogenic shock slowly improved. His postoperative course included mediastinal washouts with ultimate chest closure. Although his course was complex, including acute kidney injury requiring dialysis and heparin-induced thrombocytopenia, he made a full recovery and was discharged to cardiac rehabilitation on postoperative day 19. At 1-month follow-up, he was doing well living at home.

## Comment

This case highlights the successful surgical treatment of a patient with a history of a Freestyle ARR in whom a left SOV pseudoaneurysm developed that fistulized into a distal pulmonary valve homograft. Understanding the cause of an APF after a Freestyle implantation is critical so that serious complications can be anticipated. Although often attributed to pseudoaneurysmal aortic erosion, other factors are implicated too in the development of APFs, such as endocarditis, ruptured thoracic aortic or SOV aneurysms, Marfan syndrome, trauma, aortic dissection, inflammatory or syphilitic aortitis, and prior aortic or valvular surgery.^1^^,^^2^ There were no signs of endocarditis, and the most likely cause is a left SOV pseudoaneurysm that eroded into a pulmonary valve graft after an ARR and pulmonary homograft implantation. APFs after aortic surgery have been described, particularly APFs secondary to coronary button pseudoaneurysms after Bentall and after aortic valve and supracoronary graft replacement.^3^^,^^4^

Freestyle stentless porcine bioprosthesis valves have good long-term durability and excellent hemodynamic function, with low gradients often better than stented valve conduits. Pseudoaneurysms are a rare complication of Freestyles, the estimated incidence ranging from 1.1% to 4.7%.^5^ Previous literature attributes pseudoaneurysm formation after bioprosthetic implantation to inadequate tissue fixation with a subsequent immunologic response.^5^ Only 2 previous cases describing APFs after Freestyles have been reported. One was caused by subsequent endocarditis of a Freestyle that was implanted for the treatment of endocarditis.^2^ The second was an iatrogenic injury to the PA during Freestyle ARR.^6^

Interestingly, 2 instances of APFs after transcatheter pulmonary valve replacement in patients with prior Ross procedures have been described.^7^ The proposed cause was aneurysmal PAs contacting the suture lines of ascending aorta repairs that subsequently fistulized.^7^ The suture lines of replaced aortas have also been implicated in the formation of other APFs.^8^ Here, the multiple suture lines from 3 separate grafts may have weakened the remaining tissue, which under pressure and tension from the anastomoses contributed to aortopulmonary fistulization.

This case presents an aortic valve xenograft–to–pulmonary valve homograft APF, an APF between two implanted valves. We have not found any reports of an APF after Freestyle implantation that do not involve iatrogenic trauma or endocarditis, highlighting another unique feature: a nontraumatic, noninfectious Freestyle xenograft APF.

## References

[1] Premchand R.K., Bhaskar Rao B., Partani K. (2014). A rare case of acquired aortopulmonary fistula with bicuspid aortic valve: report of successful surgical repair. BMJ Case Rep.

[2] Khalid Y., Dasu N., Daneshvar M. (2021). An unusual case of an acquired aortopulmonary fistula after surgical replacement of a bicuspid aortic valve. Case Rep Cardiol.

[3] Ferrari G., Anastasio G., Bianchi M., Scioti G., Guarracino F., Bortolotti U. (2012). Aortopulmonary fistula after a modified Bentall procedure. J Heart Valve Dis.

[4] Maeder M.T., Wolber T., Künzli A., Genoni M., Blank R., Rickli H. (2006). Aortopulmonary fistula occurring 4 years after replacement of the ascending aorta. Ann Thorac Surg.

[5] Englum B.R., Pavlisko E.N., Mack M.C. (2014). Pseudoaneurysm formation after Medtronic freestyle porcine aortic bioprosthesis implantation: a word of caution. Ann Thorac Surg.

[6] Kameda Y., Mizuguchi K., Kuwata T., Mori T., Taniguchi S. (2004). Aortopulmonary fistula due to perforation of the aortic wall of a freestyle stentless valve. Ann Thorac Surg.

[7] Kenny D., Holoshitz N., Turner D., Hijazi Z.M. (2013). Aortopulmonary fistula after transcatheter pulmonary valve replacement. Circ Cardiovasc Interv.

[8] Mahmoud O., Elias H., Rafiq A., Alsaid A. (2020). Acquired aortopulmonary fistula: a case report. Eur Heart J Case Rep.

